# Evaluation of the dislodgement force of splinted restorations with engaging conical abutments over multiple nonparallel implants (in-vitro study)

**DOI:** 10.1186/s12903-023-02958-4

**Published:** 2023-05-17

**Authors:** Mohamed Moataz Khamis, Niveen Hazem Zakaria, Khaled Mohamed Farrag, Salma Abolgheit

**Affiliations:** 1 Department of Prosthodontics, Faculty of Dentistry, Alexandria University, Alexandria, Egypt; 2 Division of Fixed Prosthodontics, Conservative Dentistry Department, Faculty of Dentistry, Alexandria University, Alexandria, Egypt; 3 Department of Dental Biomaterials, Faculty of Dentistry, Alexandria University, Alexandria, Egypt

**Keywords:** Splinted restorations, Screw-retained restorations, Multiple nonparallel implants, Implants divergence angle, Conical implant abutment connections, Multiple engaging abutments

## Abstract

**Background:**

Splinted multiunit cement-retained restorations with screw access channels over engaging abutments are viable implant prosthetic options. However, information regarding the maximum degree of divergence between multiple implants is lacking. The purpose of this in vitro study was to determine the maximum degree of divergence between 2 adjacent implants with conical connections that allows insertion and removal of splinted restorations with engaging preparable abutments or titanium base abutments.

**Methods:**

Two implants were aligned in a stone base, one straight and the other at an angle ranging from 0 to 20 degrees. The implants represented an implant system that had an internal conical connection and a hexed abutment engaging the base of the connection. Two straight preparable engaging cement retained abutments were screwed onto the implants and splinted together using acrylic resin. A total of 11 angles were tested, with 7 specimens for every angle. Evaluation of dislodging force was performed by pulling out the splinted abutments after unscrewing them. This was performed subjectively by 3 blinded investigators who applied a tactile pulling force. A scale of 0–10 was used to estimate the pulling force. Objectively the dislodging force was measured in Newtons using a universal testing machine. A statistical correlation was made between the subjective and objective dislodging force values using Spearman’s rank correlation coefficient.

**Results:**

The mean subjective values increased gradually from 0 to 16 degrees. A sudden rise was noticed at 18 degrees (9.71 ± 0.23) and, at 20 degrees, the investigators were not able to remove the splinted abutments from the implants. The mean objective dislodgement force values increased gradually from 0 to16 degrees and abruptly from 16 degrees (13.57 ± 0.45 N) to 18 degrees (25.40 ± 0.66 N) and 20 degrees (35.22 ± 0.64). The correlation between the subjective and the objective evaluations assessed using the Spearman’s rank correlation coefficient was 0.98 indicating a statistically significant correlation (*P* < .001). As the objective dislodging force increased, the subjective dislodgement difficulty increased.

**Conclusions:**

Splinting cement retained restorations with screw access channels on engaging abutments is possible when multiple implants with conical connections having an internal flare angle of 8 degrees are used, with implant divergence of up to 16 degrees.

## Background

Splinting multiple implants by using cement retained restorations with screw access channels combines the advantages of screw and cement retention including simplicity of construction, passivity, and ability to remove excess cement [1–3]. To ensure and maintain abutment orientation, engaging abutments need to be used. However, the use of such abutments requires implants to be parallel to allow for insertion and removal of the prosthesis abutment assembly [3]. An implant abutment connection that allows a degree of implant divergence without interfering with the insertion and removal of a splinted restoration with engaging abutments is desirable [3].

Splinted screw-retained restorations are generally designed with non-engaging connections to avoid interference during insertion and removal especially with non-parallel implants [1–4]. The use of engaging components in the presence of slightly divergent implants has been reported [3, 5–7] based on the presence of a minimal degree of tolerance related to the design of the connection [8]. Connections with long internal parallel engaging areas are much less tolerant to implant deviation compared to those with short conical connections [9, 10].

Conical implant abutment connections have become popular among implant designs [10–12] as they significantly improve the stability of the connection, reducing the incidence of screw loosening [11–13]. These abutments are usually provided with an antirotational component, commonly a hex, that engages merely the base of the connection [11]. It is not intended to stabilize the abutment implant interface, but to act as an antirotational mechanism [11] especially when preparable cement retained abutments are used [3, 6].

Conical connections have an internal flare of variable degrees. This allows a multiunit splinted restoration to be inserted and removed without interference even when implants are slightly divergent. When non-engaging abutments are used, the acceptable angle of divergence between 2 adjacent implants is the sum of the internal flare angles on either side of both implants. Choi et al [5]. suggested that a divergence of 8 degrees between neighboring Astra implants, that had an 11 degree internal taper and long hexagon internal connections, was the maximum divergence that allowed passive insertion and removal of a splinted multiunit prosthesis. The authors recommended further studies to evaluate the greater divergence commonly encountered.

When multiunit restorations with all non-engaging abutments are used, the restoration relies completely on the integrity of the abutment screws [14]. The use of multiple engaging abutments is therefore desirable as it is expected to stabilize the prosthesis and reduce the stress and strain on the abutment screw [1]. This minimizes the rate of screw loosening [4, 15, 16] and, in turn, improves peri-implant soft and hard tissue health [17].

The hemi-engaging design was suggested as a compromise where only one of the abutments is engaging and the rest are non-engaging [1, 15]. This design was reported to significantly increase the stability of the restoration when compared to a completely non-engaging design [4]. To determine whether to splint restorations with all engaging abutments or to use the hemi-engaging design, AlHelal et al. [1] suggested splinting engaging impression copings on all implants on a cast and using tactile sensation to pull the splint out. If it came out freely, an all-engaging design was used. If not, then a hemi-engaging design was recommended.

Multiple studies and reports mentioned splinting multiunit implant restorations having all engaging abutments [1–3, 6–8, 18, 19]. However, the diverging angle between implants allowing this has not been determined in previous studies [4]. The purpose of the current study was to determine the maximum angle of divergence between neighboring implants allowing the insertion and removal of a splinted restoration with all engaging abutments. The relation between the force required to dislodge the restoration and implant divergence angle was evaluated subjectively by applying tactile pulling force and objectively by using a universal testing machine. The null hypothesis was that the degree of implant divergence would have no effect on the dislodging force of splinted restorations with engaging conical abutments.

## Methods

This study aimed to determine the maximum angle of divergence between neighboring implants allowing the insertion and removal of a splinted restoration with all engaging abutments. In this in vitro study, 2 implants were aligned, one straight and the other at an angle that ranged from 0 to 20. A total of 11 angles were tested (0, 2, 4, 6, 8, 10, 12, 14, 16, 18 and 20 degrees). Seven specimens, splinted bridges, were fabricated for every angle. Sample size was estimated assuming 80% study power and 5% alpha error. Lugas et al. [20] studied the influence of abutment geometry on the force needed for crown retrieval which ranged from 220 to 350 N. Based on comparison of means, sample size was calculated to be 6 per group and increased to 7 to make up for laboratory processing errors [21]. To ensure adequate power, post-hoc power was computed based on the dislodgement force at different angulations and the reported power was 98.7% (G*Power Version 3.1.9.4).

**Fig. 1:**
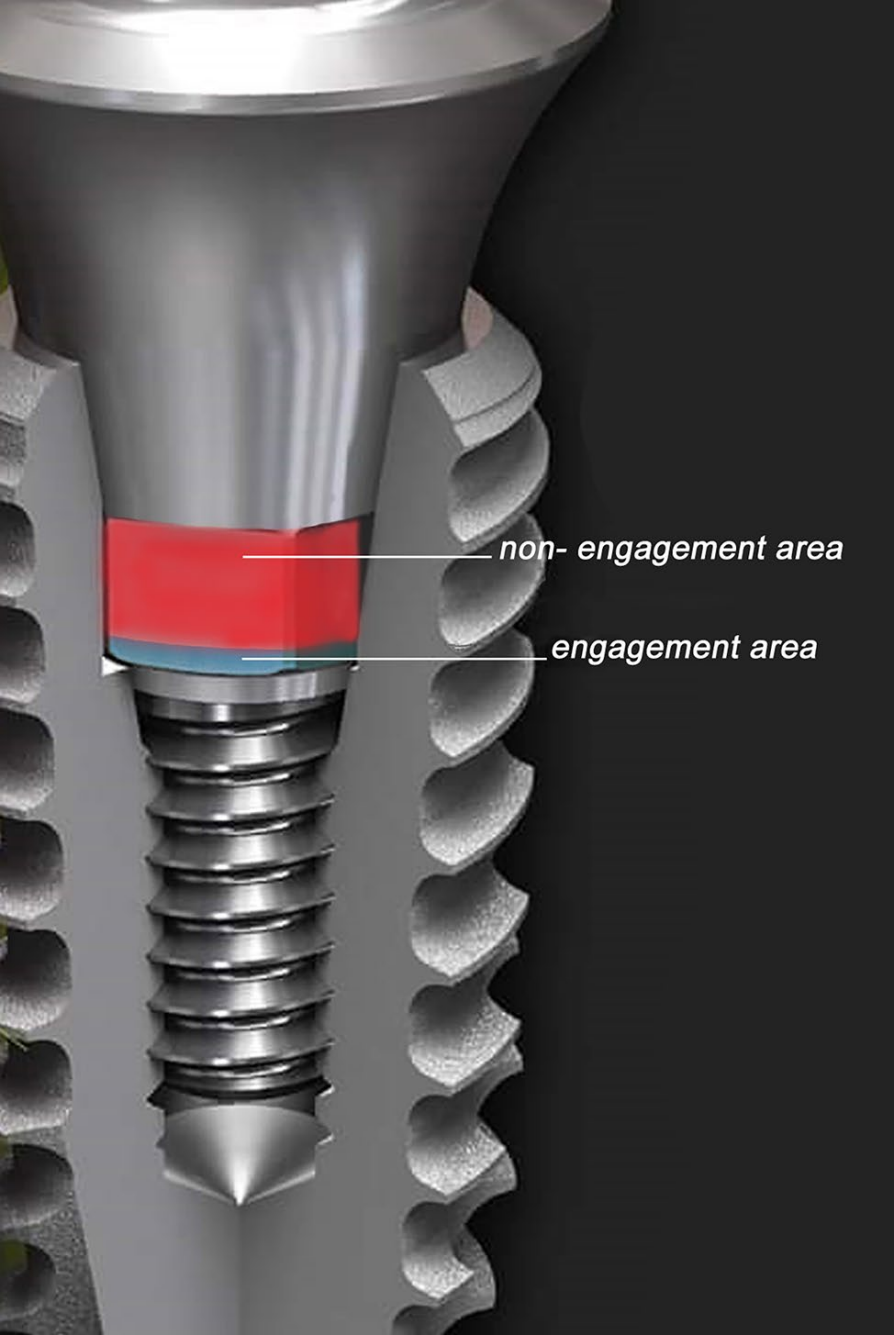
Hexed abutment engaging the base of the internal conical connection

An implant system (Dentium Superline; Dentium Co, Ltd, South Korea) was selected having an 8 degree taper internal conical connection and a hexed abutment engaging the base of the connection to provide antirotation as seen in Fig. 1. A plastic container was fabricated having 2 compartments separated by a septum. Four typodont teeth (Nissin Dental Products, Kyoto, Japan) were set in dental stone, 2 in every compartment. The root portion of the 2 medial teeth, a premolar and a molar, were lubricated to allow future detachment. A putty index (Zetaplus putty, Zhermack SpA, Badia Polesine, Italy) was made over the teeth The 2 medial teeth were removed after the stone was set and the socket of the root portions of the teeth was widened to allow the placement of 2 implants.

In each compartment, one implant was aligned by using a device fabricated to accurately position the implants at specific angles by using a dental surveyor (Ney surveyor; Dentsply International Inc., Pennsylvania, USA). The device consisted of a platform with a movable base to which a protractor was attached to measure the angle of the hinged base raised by using a screw [22].

To align the implant, the long screw of an open tray transfer coping was screwed to the implant. It was then attached to the tool holder of the surveyor. The plastic container was placed over the hinged base of the device that was set to zero tilt. Soft stone was mixed and placed in 1 compartment of the container and the implant slowly lowered into the stone until its platform was flushed with the surface of the stone. To align the angled second implant, the base was elevated by turning the bottom screw of the device until the desired angle was reached Stone was placed into the other compartment and the implant was lowered into the stone that was left to set.

**Fig. 2:**
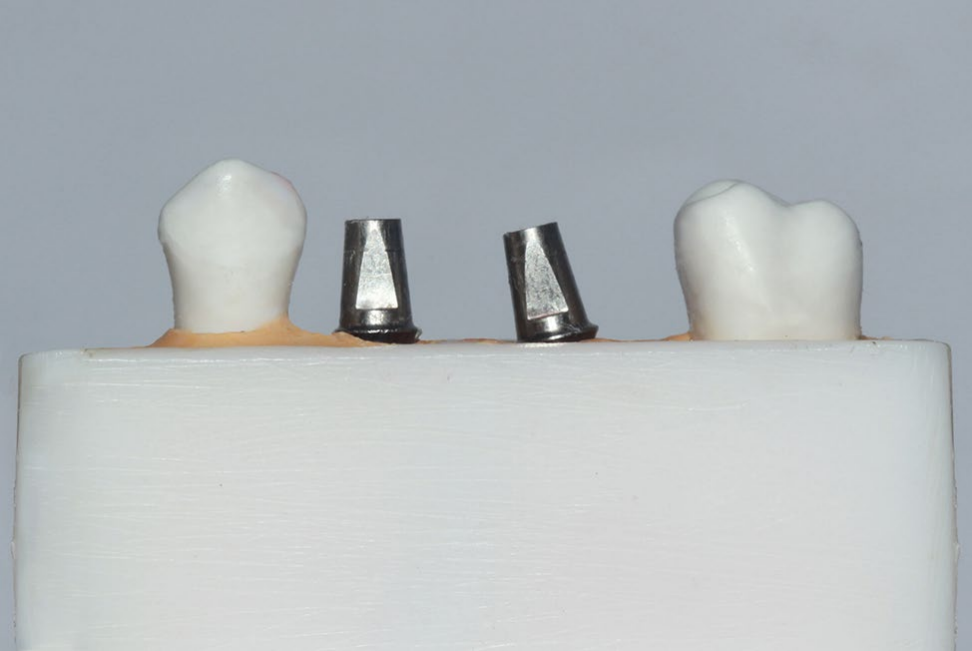
Two straight preparable engaging cement retained abutments screwed onto the implants

**Fig. 3:**
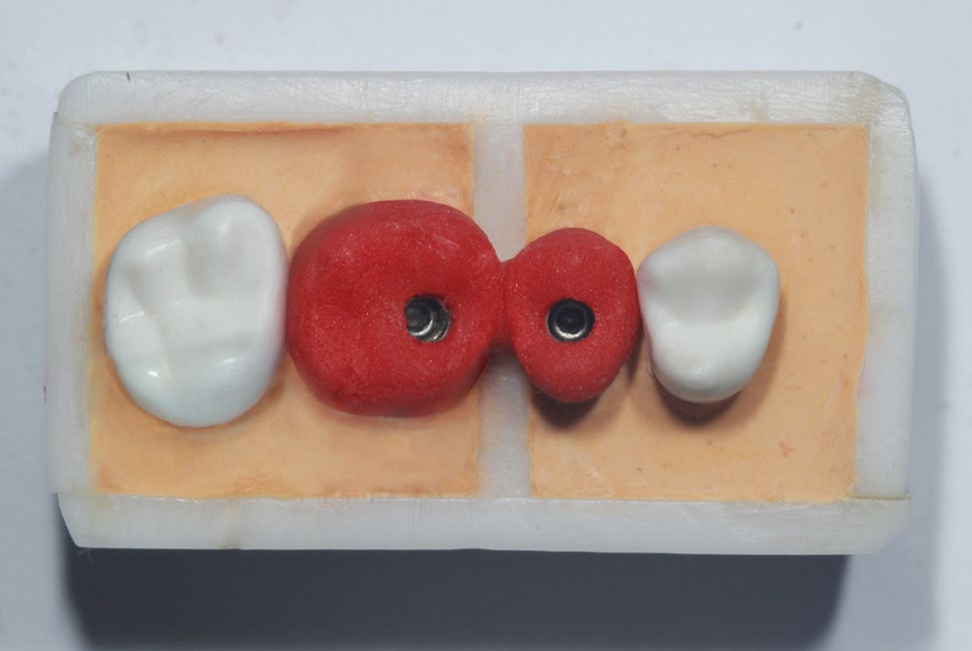
Occlusal view of the splint over the abutments

Two straight preparable engaging cement retained abutments were screwed onto the implants as seen in Fig. 2. Abutments were splinted together by using acrylic resin pattern material (Duralay; Reliance Dental Manufacturing, Illinois, USA) that was poured into the rubber base index and left to polymerize overnight. A diamond disk was used to separate the splint midway between from the 2 implants with a 0.2-mm standardized gap space. The interproximal contacts were adjusted and evaluated using 8-µm metallic shims (Shim stock; Almore International Inc., Oregon, USA) [23]. The pieces were then reconnected using the incremental technique to minimize polymerization shrinkage of the resin [5, 8]. The acrylic splint was 8 cm in length and was in contact with the ivory artificial teeth on both sides as seen in Fig. 3.

Evaluation of the dislodging force was performed subjectively by applying tactile pulling force performed by 3 blinded investigators and objectively by using a universal testing machine. For every specimen, the splinted abutments were hand tightened by the same investigator, then the screws were unscrewed and removed without any movement of the abutments over the implants. Investigators were asked to assess the dislodging force on a scale from 0–10 where zero denoted no resistance during removal and 10 denoted inability to remove the splint. Training and calibration on the subjective measurements was performed, and intra- and inter-examiner reliability was calculated. Intraclass Correlation Coefficient ranged from 0.935 to 0.986, indicating excellent agreement between examiners and across time [24].

Lab evaluation was performed for all specimens to objectively assess the dislodging force. Splints were pulled off in an axial direction by using a universal testing machine (5ST; Tinius Olsen, Surrey, England) at a crosshead speed of 1 mm/min. The force of pulling the splints out was measured in newtons (N).

Data were statistically analyzed by using a software program (IBM SPSS Statistics, v23; IBM Corp, New York, USA). The subjective and objective evaluation of the dislodging force were analyzed through calculation of the mean and standard deviations of the values. Comparisons of objective and subjective evaluation between different angles were performed using Kruskal Wallis test, followed by multiple pairwise comparisons using Bonferroni adjusted significance level. To identify a correlation between the objective and subjective evaluation, the Spearman’s rank correlation coefficient was calculated.

## Results

**Fig. 4:**
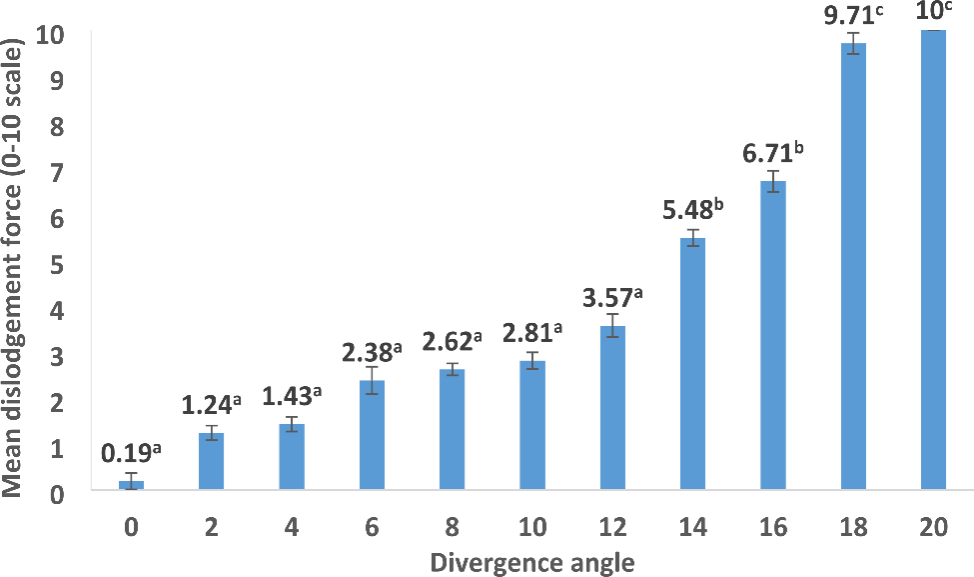
Subjective dislodgement force as reported by investigators on a scale from 0–10 (different superscripted letters denote statistically significant differences between angles)

**Fig. 5:**
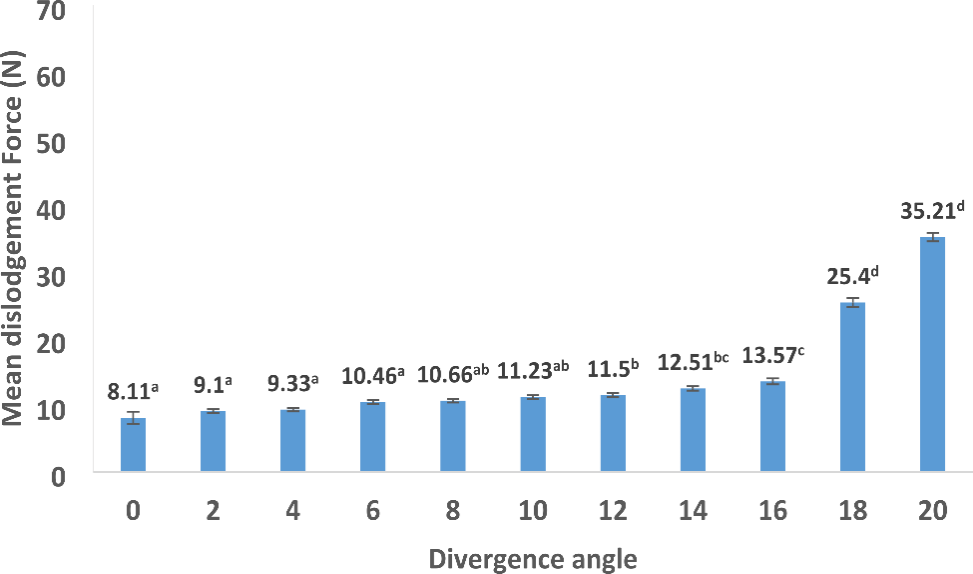
Objective dislodgement force measured by the universal testing machine in Newtons (N) (different superscripted letters denote statistically significant differences between angles)

The values of the subjective dislodging force (0–10 scale) and objective lab evaluation (N) are described in Table 1. Regarding the subjective evaluation, the mean values increased gradually from 0 to 16 degrees. A sudden rise was noticed at 18 degrees (9.71 ± 0.23) and at 20 degrees at which the investigators were not able to remove the splinted abutments from the implants (Fig. 4). The mean objective dislodgement force values (Fig. 5) increased gradually from 0 to16 degrees and abruptly from 16 degrees (13.57 ± 0.45 N) to 18 degrees (25.40 ± 0.66 N) and 20 degrees (35.22 ± 0.64).

**Table 1:** Subjective (0–10 scale) and objective (N) dislodgement force at each angle

Angle (degrees) (n = 7)	Mean ± standard deviation (0–10 scale)	Mean ± standard deviation (Newton)
0	0.19 ± 0.18^a^	8.11 ± 0.91^a^
2	1.24 ± 0.16^a^	9.10 ± 0.29^a^
4	1.43 ± 0.16^a^	9.33 ± 0.26^a^
6	2.38 ± 0.30^a^	10.46 ± 0.30^a^
8	2.62 ± 0.13^a^	10.66 ± 0.26^ab^
10	2.81 ± 0.18^a^	11.23 ± 0.27^ab^
12	3.57 ± 0.25^a^	11.50 ± 0.31^b^
14	5.48 ± 0.18^b^	12.51 ± 0.33^bc^
16	6.71 ± 0.23^b^	13.57 ± 0.45^c^
18	9.71 ± 0.23^c^	25.40 ± 0.66^d^
20	10.00 ± 0.00^c^	35.22 ± 0.64^d^
KWT P value	< 0.001*	< 0.001*

n: number of specimens

KWT: Kruskal Wallis test

*statistically significant at P value < 0.001*

^a-d^: different letters denote statistically significant differences between angles using Bonferroni adjusted significance level

**Fig. 6:**
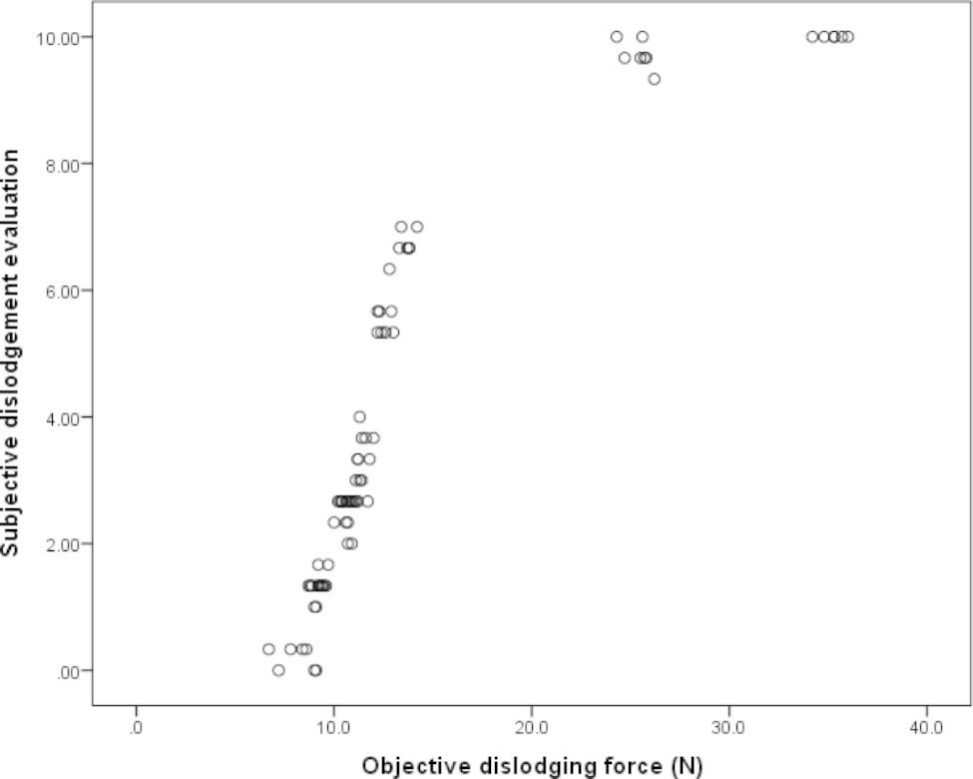
Graphic representation of the correlation between subjective and objective evaluation

When the correlation between the subjective and the objective evaluations was assessed, the Spearman’s rank correlation coefficient was 0.98 indicating a statistically significant correlation (*P* < .001) as seen in Fig. 6. The Spearman correlation coefficient takes values between + 1 and − 1; the closer the coefficient is to + 1 or -1, the stronger the association. Consequently, the coefficient of 0.98 indicated a strong direct relationship between the subjective and objective evaluations. As the subjective dislodgement difficulty increased, the objective dislodging force increased.

## Discussion

The current study aimed to determine the possibility of splinting restorations with engaging abutments on non-parallel implants and determine the maximum degree of implant divergence beyond which splinting was not possible. The null hypothesis was rejected. The degree of implant divergence had an effect on the dislodging force of splinted restorations with engaging conical abutments.

Evaluation involved the ability to dislodge the restoration by hand while estimating the difficulty and resistance involved on a scale from 0 to 10. Three investigators were involved to minimize the subjectivity. An objective test was added by using a universal testing machine to compare and confirm the subjective values with objective readings.

The use of engaging abutments in splinted restorations is preferred to improve the integrity of the abutment implant connection. Such engaging abutments can be either titanium base abutments or preparable cement retained abutments in cement retained restorations with screw access channels. Theoretically, it is not possible to use multiple engaging abutments with non-parallel implants [2, 4]. However, implants with conical connections can allow a degree of divergence depending on the degree of flare of the internal conical connection [10].

The implant system selected for the current study represents a wide group of implants with similar design. They have a hexagonal engaging component, which provides antirotation, at the base of the internal conical connection, responsible for connection integrity. Implants were used instead of implant analogues, to minimize the possibility of relative tolerance of the internal conical connection of implant-abutment interfaces if analogs were used. A divergence of up to 20 degrees was selected as it is beyond the 16-degree divergence obtained from adding the 8-degree flare of the conical connection of both implants that would, theoretically, allow the insertion and removal of a splinted restoration.

The splinted abutments were hand tightened to simulate the clinical procedure of cement-retained restorations with screw access channels. In the patient’s mouth, the restoration is cemented onto the abutments, then unscrewed together with the abutments as one unit for excess cement removal before being screwed over the implants and torqued according to manufacturer’s instructions [1, 2]. Acrylic resin was used to splint the abutments as it is a fast setting, dimensionally stable material that is easily constructed. Although it is considered a resilient material, it was made in a thick cross section to ensure rigidity and fabricated using a mold for standardization of dimensions [5]. To overcome the problem of polymerization shrinkage, the resin splint was fabricated, left overnight, then separated and reconnected just before the evaluation procedures [5, 8].

Evaluation of the dislodging force was performed by hand pulling the acrylic splint by blinded investigators. The perceived resistance was recorded on a scale from 0 to 10 by every investigator. The mean and standard deviations were calculated. The dislodging force measured by a universal testing machine was recorded to objectively justify the subjective values.

The mean values of the subjective and objective evaluations showed a gradual increase in the dislodging force between angles from 0 up to 16 degrees. A slight resistance was found in the subjective (0.19 ± 0.18) and objective values (8.11 ± 0.91) even when both implants were parallel (0-degree angle). The reason for this is that splinted abutments were hand tightened then unscrewed without dislodging them. The proper fit and precision of the conical implant abutment connection created the slight resistance.

The resistance increased as the angle between both implants increased. This can be explained by the opposing sides of the internal conical connection of the implants approaching parallelism, resulting in increased friction between the abutment and implant connection. When the angle between both implants reached 16 degrees, the opposing internal connection sides of the implants became parallel, allowing the dislodgement of the splinted abutments with resistance related to the maximum frictional force between the abutment implant interfaces.

When the angle between both implants increased above 16 degrees, at 18 degrees, a sudden increase in the subjective (9.71 ± 0.23) and objective values (25.40 ± 0.66) were recorded. The investigators were able to dislodge the splinted abutments with significant difficulty. At 20-degree angle, the investigators were not able to dislodge the splinted abutments by hand force. The universal testing machine required 35.22 ± 0.64 N to dislodge the splinted abutments. When the mean objective and subjective values were statistically correlated, a strong direct relationship between both was found confirming the clinically perceived values.

**Fig. 7:**
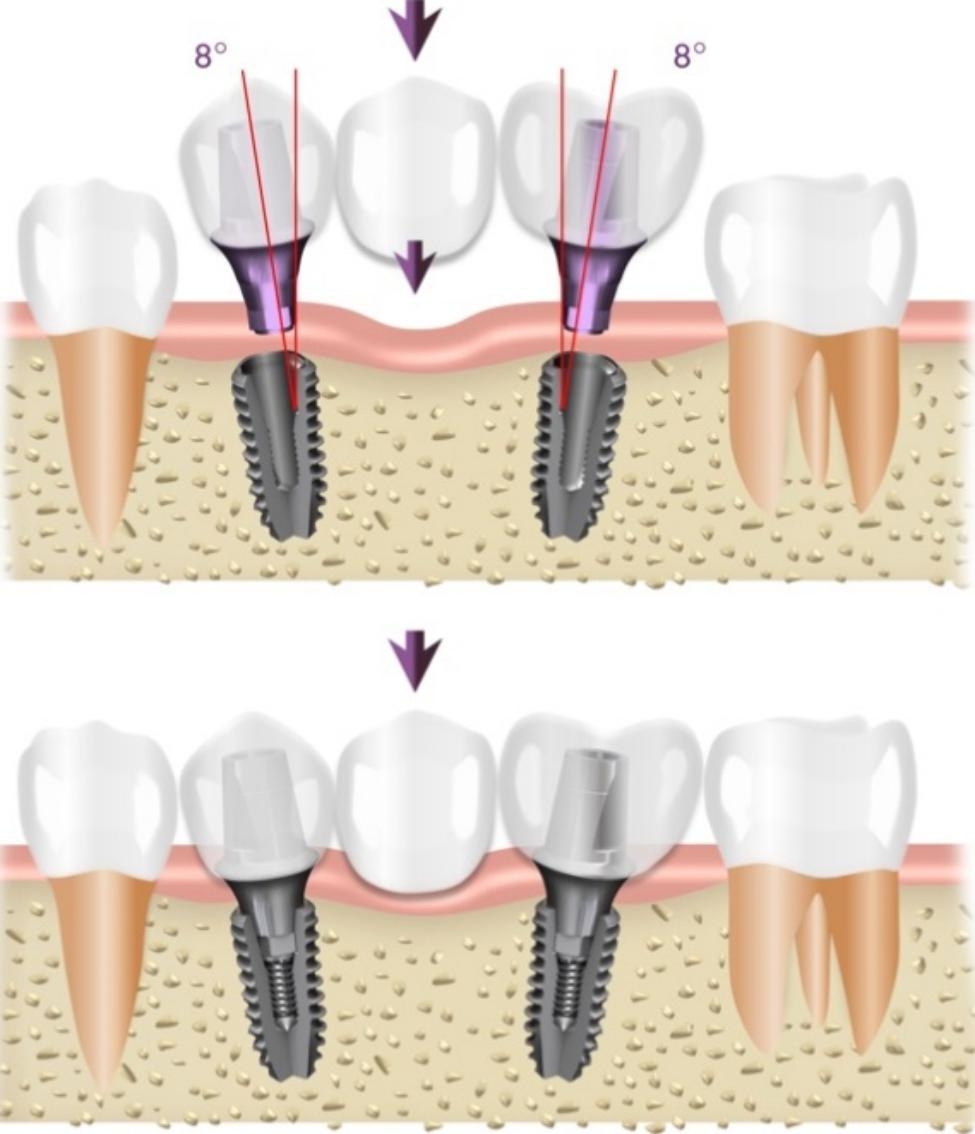
Acceptable implant divergence angle measured as the sum of internal flare angles of the conical connections

The findings of the current study indicate that splinting restorations with engaging abutments is possible with multiple nonparallel implants provided that the implants have internal conical connections and engaging components at the base of the conical connection. This design allows anti-rotation together with a degree of tolerance to accommodate a minimal amount of divergence between neighboring implants. Based on the results of this study, it appears that the acceptable diverging angle between the implants is measured by the sum of the internal flare angles of the conical connections as shown in Fig. 7.

The findings of the present study cannot be applied on other implant abutment connection designs especially those with long engaging components, which is a limitation of the study. Another limitation is that the degree of divergence has been evaluated in only a single dimension. Further studies on other implant abutment connections are recommended. A clinical study indicating the applicability of the present findings is also needed.

## Conclusion

Within the limitations of the current study, it is possible to use splinted cement retained restorations with screw access channels having engaging abutments over multiple non-parallel implants provided they have conical connections with an internal flare angle of 8 degrees, and with implant divergence of up to 16 degrees.

## Data Availability

The raw data of the present study is available at: https://figshare.com/s/eb4d0acd0b544727ddb6.
